# Deep Eutectic Solvents and Multicomponent Reactions: Two Convergent Items to Green Chemistry Strategies

**DOI:** 10.1002/open.202100137

**Published:** 2021-08-17

**Authors:** Francisco G. Calvo‐Flores, Cristina Mingorance‐Sánchez

**Affiliations:** ^1^ Grupo de Modelización Molecular Dpto. de Química Orgánica Facultad de Ciencias Universidad de Granada 18071 Granada Spain; ^2^ Grupo de Modelización Molecular Dpto. de Química Orgánica Facultad de Ciencias Universidad de Granada 18071 Granada Spain

**Keywords:** alternative solvents, biocompatible solvents, deep eutectic solvents, green chemistry, sustainability

## Abstract

One of the highlights of green chemistry is the development of techniques and procedures with low environmental impact. In the last years, deep eutectic solvents (DES) have become an important alternative to conventional organic solvents. For a period ionic liquids have provoked remarkable interest, but they have been displaced by DES because they show easier preparation methods, lower prices, many of them are biodegradable and compatible with biological systems. In addition, they show adjustable physicochemical properties, high thermal stability, low volatility and are compatible with water. In this paper is reviewed the state of the art of the use of DES paying special attention to the role of reaction media in organic synthesis.

## Graphical abstract

**Deep eutectic solvents** are an interesting alternative to conventional solvents, both as a reaction medium and for many processes and technologies used in chemistry and related sciences. They are easily prepared, versatile, many of them can be obtained from renewable sources and can be designed and adapted for specific processes. For all these reasons, they have been studied intensively in recent years and their interest is constantly growing. This paper reviews some of the most prominent applications and which are the fields of interest that may be a focus of applications in the near future.

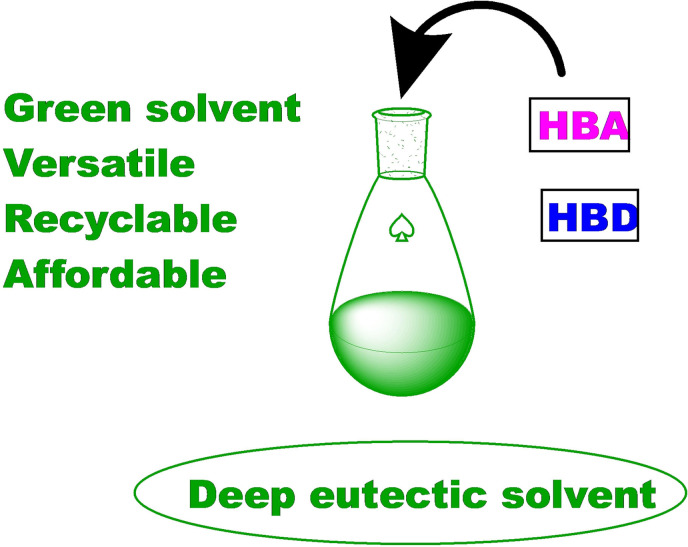

## 1 Introduction

The evolution of synthetic chemistry is not unconnected with the social concern about a more sustainable economy^[1]^ and an environmentally friendly society. Green chemistry^[2]^ is the ethical response from chemical sciences to such concerns. Synthetic classic procedures in chemistry, used to show high efficiency but their environmental impact has been taken into consideration as a key factor only when green chemistry arises. Green chemistry aims to develop more sustainable methodologies and procedures, with less environmental impact, minimizing risks to professionals and the general public in contact with all kinds of chemicals.^[3]^

Green chemistry is focused on the development of new synthesis methods to avoid negative impacts on human health and the environment. Criteria such as atom economy,^[4]^ as an efficiency parameter, avoiding the formation of by‐products, replacing risky reagents or catalysts with less harmful substances, and energy efficiency of the processes. Such conditions provide the design of chemical reactions under a green chemistry perspective. In the last decades a remarkable effort has been made to replace many organic solvents commonly used in industry and laboratories for others safers, less toxic with a lower impact on the environment and when it is possible obtained from renewable sources. Deep eutectic solvents are a set of new solvents that may be prepared from a combination of solids compounds that become liquids when mixed and heated, and may be used as solvents for example, in chemical reactions, or extraction of high value compounds from biomass.

## 2 Conventional Organic Solvents Versus Deep Eutectic Solvents

Conventional organic solvents are carbon‐based compounds. Most of them show high vapor pressure values and most of them are water immiscible. They constitute a heterogeneous group of volatile compounds chemically diverse and mainly obtained from the petrochemical industry. Organic solvents are widely used as reaction media, but in many other industrial branches and household products.^[5]^

Most conventional organic solvents show serious risks and hazards for handling, storing or using them because they use to be flammable, explosive, and they may form compounds with high toxicity when they break down at high temperatures. They produce harmful health effects when they come into direct contact with mucous, skin or eyes,^[6]^ and by inhalation they are distributed quickly into the blood and vital organs.

From the point of view of environmental chemistry, most of organic solvents are considered as volatile organic compounds,^[7]^ so they may be emitted to the atmosphere and contribute to atmospheric pollution participating actively in many photochemical processes,^[8]^ that produce the photochemical smog and some of them, as the very well known halogenated solvents contribute to ozone depletion.[^7^, ^9^]

To avoid the inherent risks and hazards related to the massive use of solvents in laboratories and industry in the last years, a lot of work has been done in two main directions:

1. The publication of several guides and recommendations for the substitution of solvents with a high impact on human health and the environment, or potentially dangerous because of their flammability, or explosion risks[^10^, ^11^] by others less hazardous solvents.
2. The introduction of non‐conventional solvents as a general practice in laboratories and industrial processes,^[12]^ paying special attention to those biodegradable and obtained from renewable sources.[^13^, ^14^]

With this background there is a strong tendency to replace most conventional organic solvents with others, with lower environmental impact obtaining the same results.

A green alternative to conventional organic solvents is the so‐called Deep Eutectic Solvents (DES).[^15^, ^16^, ^17^] DES are obtained by mixing together two or more solid substances, to give an eutectic mixture, showing significant negative deviations to thermodynamic ideality in the liquid state.^[18]^ They may contain a variety of organic and inorganic charged species (anionic and/or cationic) and they use to be cheap, renewable, and biodegradable, The term DES has been coined mainly to differentiate them from another family of non‐conventional solvents, the so called ionic liquids (IL). IL are another class of non volatile salt‐like solvents that were reported for the first time by Walden in the early years of the 20th century.^[19]^ Both IL and DES may be prepared from organic and/or inorganic cations and anions, but DES can also be obtained from non‐ionic molecules, and therefore are not considered true IL.[^19^, ^20^] Table 1 shows the main remarkable differences between both types. A critical difference is that DES are actually environmentally friendly compared with IL.

**Table 1:** Differences between DES and IL.

Deep Eutectic Solvents	Ionic Liquids
Biodegradable and nontoxic	Not always environmentally friendly, they can be toxic
High conductivity	Conductivity‐moderate to high
Very affordable	Expensive: recycling is critical
Viscosity can be lowered by mixing with suitable ionic solvents	High viscosity
Simple synthesis by mixing inexpensive starting components. No subsequent purification is required	Complex synthesis and purification
Generally, not moisture sensitive and water compatible	Moisture sensitive must be handled under dry or inert conditions

DESs are prepared by a combination of two or more solids to give a mixture which often has a melting point very close to room temperature. They use to exhibit low toxicity and volatility, a remarkable thermal stability in a wide range of temperatures, low volatility, low vapor pressures, and tunable polarity and most of them are biodegradable. Another characteristic is that they are easy to prepare, and inexpensively. The starting material may be inorganic compounds, organic materials from the petrochemical industry and even from renewable sources. This last group is nominated as Natural Deep Eutectic Solvents (NADES).[^21^, ^22^, ^23^]

The more accepted classification of DES divides them into four groups:^[24]^

- Type I:They are formed by a quaternary ammonium salt and a metal chloride and may be considered analogous to metal halide/imidazolium salt systems. This is a group that has been described before in literature.^[25]^
- Type II: They are prepared from a quaternary ammonium salt and metal chloride hydrate.^[26]^ The relatively low cost of many hydrated metal salts and their inherent insensitivity to air/humidity makes their use in industrial processes viable.
- Type III: They are composed of quaternary ammonium salt and a hydrogen bond donor HBD.^[27]^ In Type III, choline chloride and HBDs have been widely used for many applications, such as metal extraction and organic synthesis.
- Type IV: They are composed of metal chloride and HBD. These liquids are simple to prepare and relatively unreactive with water; many are biodegradable and have a relatively low cost.^[28]^ Table 2
  **Table 2:** Classification of DES and examples.

Type	Formula	M/Z	Examples
Type I – Organic salt/inorganic salt	Cat^+^X^−^+ zMCl_x_	M=Zn, Sn, Fe, Al, Ga, In	ChCl/CoCl_2_
Type II – Organic salt/hydrate inorganic salt	Cat^+^X^−^/zMCl_x_yH_2_O	M=Cr, Co, Cu, Ni, Fe	ChCl/CoCl_2_.6H_2_O
Type III – Organic salt/hydrogen donor	Cat^+^X^−^/RZ; MClx/RZ	M=Al, Zn; Z=CONH_2_, OH, CO_2_H	ChCl/Urea
Type IV – Al, Zn chloride/hydrogen donor	MCl_x_/zRZ	M=Al, Zn; Z=CONH_2_, OH	ZnCl_2_/Urea
shows some examples of DES of every type.

To this classic classification a new type of DES type has arisen recently, the so called Type V DES formed from non ionic species that may improve potential recovery and regeneration of the eutectic by evaporation,^[29]^ which implies a significant advantage over ionic DES.

The interest in DESs is increasing every year and the number of publications devoted to DESs from 2004 to the present has an unstoppable progression. Table 2 shows some examples of DES of every type.

## 3 Preparation and Properties of DES

**Figure 1:**
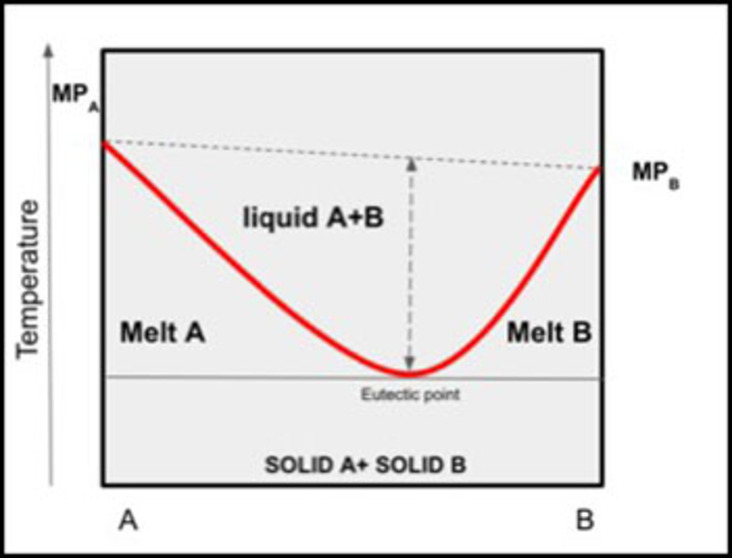
Melting diagram of a binary DES.

Deep eutectic solvents are prepared with mixtures of solid substances that can be described as Lewis or Bronsted acids and bases. One component acts as a hydrogen bond acceptor and other as a hydrogen bond donor. A variety of anionic and/or cationic species may be used as starting material for preparing a DES. When mixed, those compounds have the peculiarity of having a lower melting point than the species that form them. Thus, through a thermal equilibrium process, two solid substances form a liquid phase at their eutectic temperature^[30]^ (Figure 1).

They are prepared very easily, adding an appropriate amount of solids in a flask, then heated and stirred until a colourless liquid. is formed. The experimental procedure may be accelerated with ultrasounds^[31]^ microwaves.^[32]^ The procedure is very simple and does not produce by‐products, so it may be considered an environmentally friendly synthesis since there is no waste and no emissions and the atom economy^[4]^ is 100 %, since all the initial atoms are included in the final mixture. When used as reaction media, the high solubility of DES in water allows the separation of organic products as an insoluble layer in water with the addition of some water that dissolves the DES, avoiding the typical extraction of organic solvents at the end of the reaction. DES can be recovered by evaporating water from the aqueous layer to obtain again the starting DES. The molar ratio corresponding to the eutectic point is variable in composition and also in temperature according to the nature of each component.^[33]^ So it is possible to obtain several DES types with the same components. Binary DES are the most studied and used systems but the design and synthesis of ternary DES[^34^, ^35^] allows to expand their use and applications.

**Figure 2:**
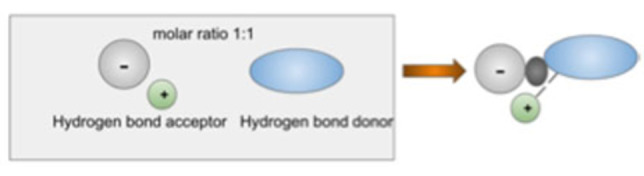
Schematic Synthesis of DES.

Figure 2 shows schematically how binary DES mixing an inorganic salt and organic hydrogen donor is synthetized, in this case mixing inorganic salt and organic hydrogen bond donors.

Traditionally, DESs behaviour has been rationalized using Hole theory^[36]^ for predicting most of the physicochemical properties of DES. When mixed, an amorphous structure is formed with weak forces between moieties separated by holds.[^37^, ^38^] In the case of DES with organic ions as choline chloride and a hydrogen bond donor as urea, Hammond et al., had suggest that choline interacts very strongly with chloride by hydrogen bonding. This leads to the formation of a complex ion as a most likely 3D configuration, involving one choline, one chloride and two urea molecules.^[39]^

One of the most well known examples of DES is the eutectic formed by choline chloride and urea (ChCl/Urea)in a 1 : 2 mol : mol ratio.^[40]^ This DES shows a melting point of 120 °C, while both components, separately, have melting points of 302 °C and 133 °C, respectively.

Examples of deep eutectic solvents with differet molar ratios and are given in Table 3.

**Table 3:** Examples and melting point (°C) of HBD, HBA, and DES.

HBA	HBD	MP HBA	MP HBD	HBA : HBD	MP Eutectic [°C]
ChCl	Urea	300	133	1 : 2	12,0^[41]^
ChCl	Glycerol	300	17,8	1 : 2	−40,0^[36]^
ChCl	Glycerol	300	17,8	1 : 3	1,7^[42]^
ChCl	Benzoic acid	300	122	1 : 1	95,0^[43]^
ChCl	Oxalic acid	300	190	1 : 1	34,0^[43]^
ChCl	Xylitol	300	96	1 : 1	Liquid at r.t
MePPh_3_Br	Glycerol	232	17,8	1 : 2	4,0^[44]^
MePPh_3_Br	Glycerol	232	17,8	1 : 4	15,6^[44]^
MePPh_3_Br	Glycerol	232	17,8	1 : 5	50,0^[44]^
MePPh_3_Br	Ethylene glycol	232	−12,9	1 : 5	−46,0^[44]^

Melting points (MP) in °C of the starting materials (HBD and HBA) and the resulting deep eutectic mixture are described.

Natural Deep Eutectic solvents^[45]^ are another category of DESs which give a step forward to improve the sustainability concept. This new class of deep eutectic solvents are metal free materials because they are prepared from natural sources of biocompatible substances with low cytotoxicity. They have a promising future in fields such as electrochemistry, organic synthesis, biocatalysis, extraction in foodomics, and several biomedical applications. They are composed of two or more biocompatible organic molecules, generally bio based primary metabolites.[^21^, ^22^] For example, choline chloride has been combined with many carbohydrate‐type components such as glycerol, xylitol, sorbitol, glucose, sucrose or maltose in different molar ratios.^[46]^ to provide a proper media for natural product extractions.

## 4 Physical Properties of DESs

Deep eutectic solvents show remarkable physicochemical and thermal properties, which can be easily adjusted to the desired experimental conditions. Their properties are a consequence of their weak lattice energies between their components caused by hydrogen bond interactions at room temperature, so they have a low melting point, low volatility, vapor pressure, high thermal and chemical stability, and they are capable of solubilizing a great variety of substrates.

### 4.1 Melting Point

As mentioned above, DESs are formed by mixing two solids that generate a new liquid Interaction of components that provide lower MP that of individual constituents. This decrease in freezing point comes from an interaction between HBD and salt. All known DESs melting points show up lower than 150 °C. It should be noted that the number of DES that are liquids at room temperature is still quite limited. Taking into account the different types of eutectic solvents, some trends can be highlighted.

In Table 3 it can be observed that for a given species HBA (ChCl) it is important to properly choose the HBD to obtain DESs with a given MP_,_ so that when using carboxylic acids such as HBD, liquid DES can be obtained at room temperature. Also, for a given HBD (urea), the nature of organic salts and the molar ratio among HBA:HBD species can significantly affect the MP of DES.[^47^, ^48^, ^49^] The presence of water, a common fact for many experimental procedures, modified melting point data, occurs for choline chloride/urea systems and should be taken into account for any physico‐chemical characterization as well as for applications of this type of solvents.^[50]^

### 4.2 Density

The density of most DES is between 1.0 g/cm^3^ and 1.35 g/cm^3^ to 25 °C, which is higher than the water density. The organization of molecules or the packaging of the HBA and HBD species, cause deSs to have higher or lower densities. DESs contain “gaps”, so that if they increase, they produce a decrease in density. On the other hand, the molar ratio of the HBA:HBD species, and the temperature, also have a significant effect on the density of these mixtures. The density of DES is influenced by both HBA and HBD and decreases with increasing temperature, which is similar to ordinary solvents.^[51]^ Some work has been published in order to predict the density of mixtures according to composition and temperature.^[52]^ These data may be of interest to optimize conditions for certain experiments.

Table 4 shows the densities of different DESs at 25 °C. Depending on composition and proportions, slightly different values are obtained.

**Table 4:** Densities of selected DESs at 25 °C.

HBA	HBD	HBA : HBD^[a]^	Density [g L^−1^]
ChCl	Glycerol	1 : 1	1,16^[36]^
ChCl	Glycerol	1 : 2	1,19^[53]^
ChCl	Glycerol	1 : 3	1,20^[36]^
ChCl	Urea	1 : 2	1,21^[54]^
ZnCl_2_	Urea	1 : 3,5	1,63^[55]^

[a] Molar relationship.

### 4.3 Viscosity

The viscosity of DES is normally high (>100 cP), however some exceptional values may be observed as ChCl‐EG (19–37 cP). In general, the viscosities of eutectic mixtures are mainly affected by the chemical nature of DES components (salt nature and HBD, molar salt/HBD ratio, etc.). The high viscosity of DESs is often due to:

- The presence of a large interaction of hydrogen bonds between the components, leading to less mobility of free species within the DES.
- Electrostatic or van der Waals interactions.
- Large ion sizes and small empty volumes of most DESs.

Viscosity is mainly controlled by volumetric factors despite strong intermolecular interactions involved in DESs. Therefore, although ion‐HBD interactions play an important role in the viscosity of DES, we take into account the serum effects. Some work has been done to provide theoretical models to predict viscosity. For example, Mjalli et al. proposed a viscosity model for choline chloride‐based mixtures,^[56]^ or based on data banks covering 27 and 156 deep eutectic solvents of different natures.[^57^, ^58^]

### 4.4 Ionic Conductivity

DES viscosity and ion conductivity are closely related. They usually have low conductivity (less than 1 mS cm) at room temperature, due to the high viscosity of most DESs. Conductivity increases with the increase in temperature and, considering that the molar ratio of the HBA:HBD species significantly influences the viscosity of DESs, this also significantly affects the conductivity of these solvents. The knowledge of this property is a key factor for electrochemical applications of DESs. Some theoretical studies have been developed recently to predict conductivity.[^59^, ^60^]

### 4.5 Polarity

DESs are considered organic alternative solvents to common volatile organic solvents. The polarity associated with DESs is an essential data if they are to be used as green alternatives to common organic solvents in industries capable of dissolving or extracting properly organic molecules.^[61]^ or however, it can be said that ChCl/Urea DESs (1 : 2), ChCl/Glycerol (1 : 2) and ChCl/Ethylene glycol are highly polar fluids and their polarity is even greater than that of short‐chain alcohols and more common ionic liquids.^[62]^

### 4.6 Surface Tension

Surface tension is generally expected to follow viscosity‐like behavior, as it depends on the strength of HBA:HBD interactions. In all DESs studied to date, surface tension increases with a decrease in temperature and molar salt fraction due to weakening of HBD. Experimental data has been recovered on a significant group of DESs[^63^, ^64^] and theoretical models have been developed to predict surface tension of them.[^63^, ^65^] This property has significant effects, for example, on permeability and bubbling, that may be considered in the design of many processes.

### 4.7 Toxicity and Biodegradation

There are two fundamental questions to talk about the green character of a solvent: toxicity and biodegradability. The first item directly affects people involved in the handling and use of these substances or the trace presence in certain products that have been manufactured with DESs. The second one is related to the entire lifetime of the stuff.

The first remarkable idea is that available toxicity data of DES are considerably less as compared to those of ionic liquids.^[66]^ Using a mtk‐QSTR model for predicting the toxicity and antimicrobial properties of several DES against various biological species, a particularly good overall accuracy and predictivity has been obtained with an external validation set.

With the possible application of DES as routine solvets in the cosmetic^[67]^ and pharmaceutical sectors,^[68]^ some studies have been made to evaluate the response of human cells exposed to DES. Such responses in terms of cytotoxicity are still very incomplete and it is difficult to obtain solid conclusions.^[69]^

In the case of NADESs because of the natural origin of its starting materials, they are generally less toxic than DES^[70]^ and much of them are considered biodegradable. All starting components of NADESs are easily metabolized by different organisms in nature. but numerous studies indicate that, depending on the choice of components used for their preparation, they also possess a certain degree of toxicity, and some of them cannot simply be considered “rapidly biodegradable”. For all this, in order to make the most of these new types of solvents and expand their applications, it is necessary to understand in depth the mechanisms of DESs and NADESs in their biotoxicity and biodegradation, to look for more components with superior biosecurity and biocompatibility, and to create a database of DESs and toxicity for their biodegradation.

## 5 Application of DES

Deep eutectic solvents can be effectively adapted and designed to provide the appropriate properties for many applications of interest creating specific mixtures for each case. The variety of HBA and HBD species combined with the different molar proportions provide countless possibilities to use them in many areas such as catalysis, organic synthesis, dissolution, purification and extraction processes, electrochemistry, new materials chemistry etc. A subclass of traditional DES are referred as hydrophobic DES (HDESs), to resolve the issue of instability in contact with water. The HDESs have been extensively studied for the isolation and extraction of volatile fatty acids, heavy metals, and bioactive compounds from aqueous solutions.^[71]^

In the next paragraphs some applications of DES in several key fields of relevance will be described.

### 5.1 Synthesis and Purification of Fuels

Deep eutech solvents have facilitated the process of biodiesel synthesis[^29^, ^72^, ^73^, ^74^] and purification.[^75^, ^76^] A key factor of biodiesel synthesis is to successfully carry out the separation of glycerol and base traces from a biodiesel from crude reaction The most common DESs for this purpose are ChCl/2,2,2‐trifluoroacetamide, ChCl/Glycerol and ChCl/Ethylene glycol. FOr example, With DES formed by ChCl/2,2,2‐trifluoroacetamide, biodiesel is produced as a major product, with a high performance, and on the other hand DES along with impurities of the reaction.^[74]^

In the field of fuel purification DESs has been tested in the extraction of aromatic hydrocarbons from reformer and pyrolysis gasolines with several choline chloride‐based DES and different hydrogen bond donors. This procedure could work at moderate temperatures at a bulky scale in this way.^[77]^ In this field, preliminary investigations have been carried out with DESs to remove sulfur compounds from fuels.^[78]^

### 5.2 Graphene Exfoliation

One of the main applications presented by DESs is the exfoliation of graphene.[^79^, ^80^] Graphene is usually obtained from adhesive tape placed on graphite and other synthesis methods based on graphite oxidation. However, these methods have great disadvantages, since with these methods graphene is obtained with structural defects and, in addition, require energy treatments with high toxicity, so it has led to the need to use new methods.

The use of DES for exfoliating graphene has been very useful, presenting important advantages, the most important to highlight is that you get a product (graphene exfoliated) of high quality because, throughout the process, there is no chemical transformation, in addition this process is simple, which facilitates the synthesis method and the estimated reaction time. The DES used is formed by choline chloride and ethylene glycol in a molar ratio of 1 : 2, which provides low melting points and low viscosity. All of this makes the Graphene synthesized exfoliated, from this method, is compatible with the elaboration of polymeric composite materials, which are mostly used for energy storage, in addition to their use in the electronics, optical and catalysis industries.

A recent example of DES application in this area is the liquid ball milling exfoliation technique used for the preparation of defects‐free multi‐layer graphene samples in choline chloride‐urea system.[^42^, ^81^]

### 5.3 Recycling Polymers

Nowadays there is a huge market for products made of polymeric plastics. They have great advantages, such as their low cost and easy manufacturing, however, they have a serious environmental impact, in two senses:

1. Most of them are produced from the petrochemical industry (non‐renewable sources).
2. Once the useful life of the plastic‐made object is finished, many polymers are hardly biodegradable.

There is, opportunity to explore polymer synthesis and recycling in environmentally friendly, renewable solvents as DES.^[82]^

**Scheme 1:**
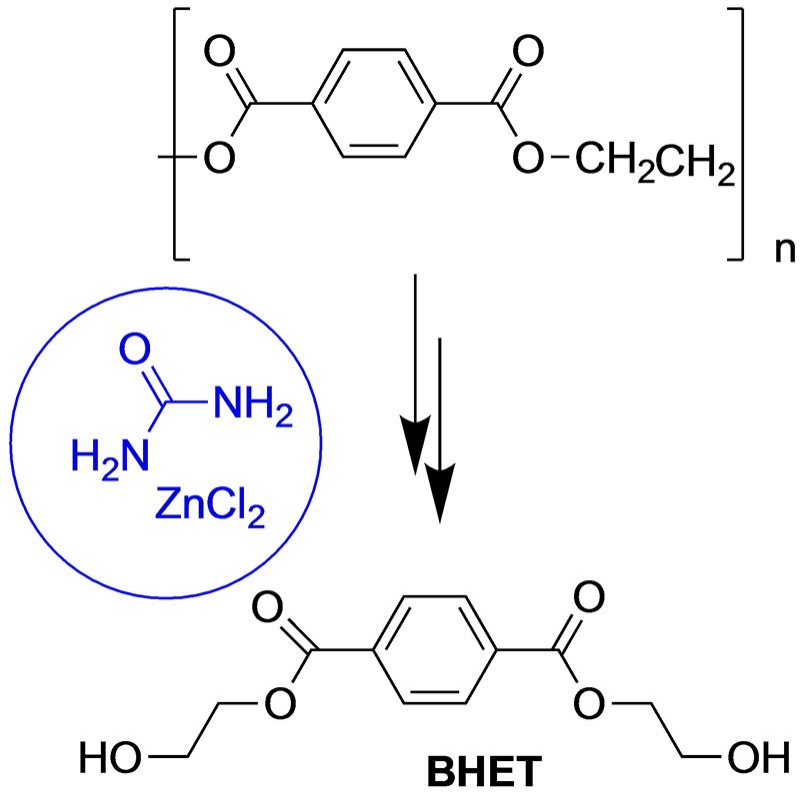
PET degradation.

Polyethylene terephthalate (PET) is of the most commonly used thermoplastic polymer used to prepare fibers, packaging, bottles for water and carbonated soft drinks etc.^[83]^ Recycling process has taken on great importance, to obtain again high value products from waste matter by depolymerization of polymer into its monomers, in order to obtain the former polymer (Scheme 1).

To achieve this reaction it is necessary to carry out the hydrolysis of the polyester chain in basic or acidic media. The DES formed by ZnCl_2_/Urea procures by itself the acidic media and simultaneously, the interaction between hydrogen of the hydroxyl group and the oxygen of urea molecules, increases the nucleophilic character of oxygen.[^84^, ^85^] The coordination of PET carboxylic oxygen with zinc promotes electrophilia. PET degradation gives bis terephthalate (2‐hydroxyethyl) monomer (BHET) which is very useful for new syntheses of unsaturated polyester and PET resins.

### 5.4 Preparation of Antibacterial Materials

Some studies have been carried out to evaluate the application of DES with antibacterial activity. Two examples of these interesting properties can be found in literature in two directions.

- The intrinsic antibacterial properties of DES
- Their use of DES in formulations of different materials to take advantage of DES as biocides

The antimicrobial activity of the formulations based on capric acid and lauric acid formulations have shown a relevant antimicrobial activity towards the gram‐positive bacteria.[^74^, ^86^] Such properties provide a great potentiality of DES as effective substitutes of currently applied infection prevention/treatment protocols.

According to the second item it has been described in literature the preparation of benzalkonium chloride (BC) and acrylic acid (AA) mixtures form a deep eutectic with antibacterial activity, that incorporated into dental resin composites procure antibacterial dental materials with better biocompatibility and long and long durability.^[87]^

### 5.5 Synthesis of New Materials

In new material manufacturing, DESs have great advantages over traditional organic solvents and ILs.

- Its preparation is simple and inexpensive.
- They are environmentally friendly.
- They are not water‐reactive, making it easy to store.
- Its ability to generate new structures by developing various functions simultaneously (solvent, catalyst, precursor or supplier of chemical species or molecules, managing agent of structure, reagent for the formation of crystalline lattices, etc.).

All this gives rise to a wide range of possibilities in the synthesis of materials assisted by DESs. Some recent publications have been done, for example, in the synthesis of nanoparticles (NPs),[^59^, ^88^, ^89^, ^90^] MOFs (organo‐metallic structures)[^91^, ^92^] and structured porosity carbons,[^93^, ^94^] DESs contribute to supramolecular organization many new materials based on the formation of hydrogen bridges between the reactions components, providing a proper environment that helps to control shapes and sizes, stabilizing agents throughout the process, etc. which is essential for the production of materials with different physicochemical and functional properties.^[95]^

### 5.6 Carbon Dioxide Fixation and Removing

CO_2_ emissions are an important environmental concern. DESs have been studying very affordable systems for CO_2_ absorption. A series of DESs with various hydrogen‐bonding donor‐acceptor pairs are being tested as CO_2_‐capturing solvents.[^96^, ^97^]

Based on solubility values of CO_2_ recovered in literature, Choline chloride/urea DES is a potential medium to resolve another problem such as efficient CO_2_ removal in natural gas and flue gas separation processes replacing the current amine‐based absorption technology.^[98]^

### 5.7 Extraction and Separation of Bioactive Compounds with DES

DESs are gaining relevance for extraction of many molecules from natural sources with several purposes: obtaining raw materials, analytic procedures, separation and purification techniques etc. They may be considered as an incipient alternative to classical extraction or separation methods with organic solvents.

Many value‐added products must be obtained from biomass but not directly. Some pre‐treatments are necessary to dissolve biomass polymers, mainly cellulose and lignin, as a previous step to convert biomass into high value‐added fuels and chemicals.[^99^, ^100^] DESs are capable of performing those pretreatments.^[101]^ In the case of lignin extraction from biomass, choline chloride‐based DES has shown remarkable efficiency in the separation of hemicellulose.

**Figure 3:**
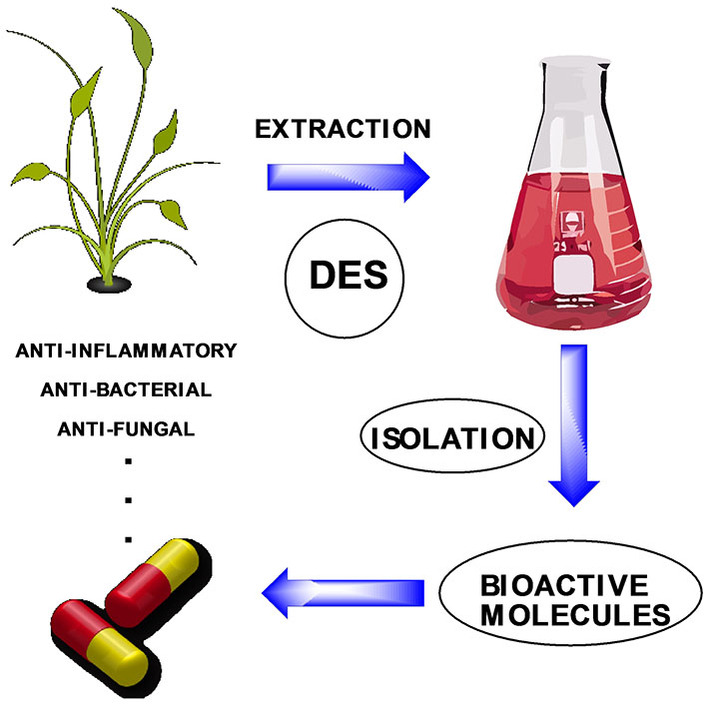
Schematic representation of extraction of bioactive molecules with DES.

Because of DESs physicochemical properties, biodegradability, low toxicity and lower price, DESs may be used to extract specific bioactive compounds, such as flavonoids,^[102]^ phenolic acids^[103]^ and polyphenols^[104]^ from various types of natural sources, favoring their synthesis and production, for use as antioxidant, anti‐inflammatory, antibacterial, etc. (Figure 3).

It can be found in literature many specific examples about the use of DES in sample microextraction preparation,^[105]^ the extraction and separation of natural products,^[106]^ bioactive compounds,[^106^, ^107^] food analysis,^[108]^ protein partitioning,^[109]^ lignin separation^[110]^ or obtention of cellulose nanocrystals.^[111]^

In the pharmaceutical industry, DESs are being taken into account for the development of methods for the extraction, separation and delivery of bioactive compounds.[^68^, ^112^, ^113^]

In addition to their use in the pharmaceutical industry, DESs shows a promising future for the production of agrochemicals,^[114]^ cosmetics^[67]^ and nutraceuticals.[^67^, ^115^]

## 6 Organic synthesis in DESs

Many organic reactions have benefited from the versatility of DESs, that procure an ideal reaction media for reactions and sometimes plays a double role because the presence of metallic salts givethem characteristic acid catalysts simultaneously.[^116^, ^117^]

A summary of some of the common reactions in organic synthesis that have been performed with DESs are outlined below. Such description may give an idea about the stay of the art of organic synthesis using DESs.

### 6.1 Redox Reactions

**Scheme 2:**
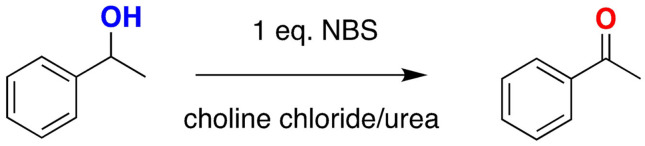
Oxidation of alcohols with NBS in choline chloride urea.

Redox reactions are reactions frequently used for functional groups interconversion. Oxidation of alcohols to aldehydes or ketones is a can be performed in choline chloride/urea at room temperature or at 60 °C with NBS, with the added value that selective oxidation of secondary alcohols in the presence of primary alcohols can be achieved^[118]^ (Scheme 2).

**Scheme 3:**
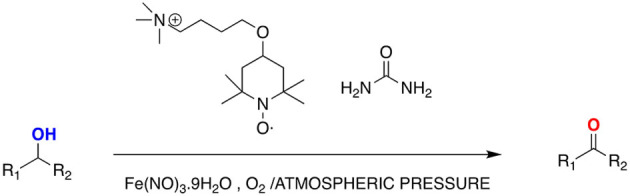
Oxidation of alcohols in DES‐TEMPO system.

Deep eutectic solvent supported TEMPO (DES‐TEMPO) composed of *N*,*N*‐dimethyl‐ (4‐(2,2,6,6‐tetramethyl‐1‐oxyl‐4‐piperidoxyl) butyl)dodecyl ammonium salt and urea constitute an efficient catalytic system for the oxidation of alcohols to aldehydes or ketones with molecular oxygen^[119]^ without any other solvent (Scheme 3).

**Scheme 4:**
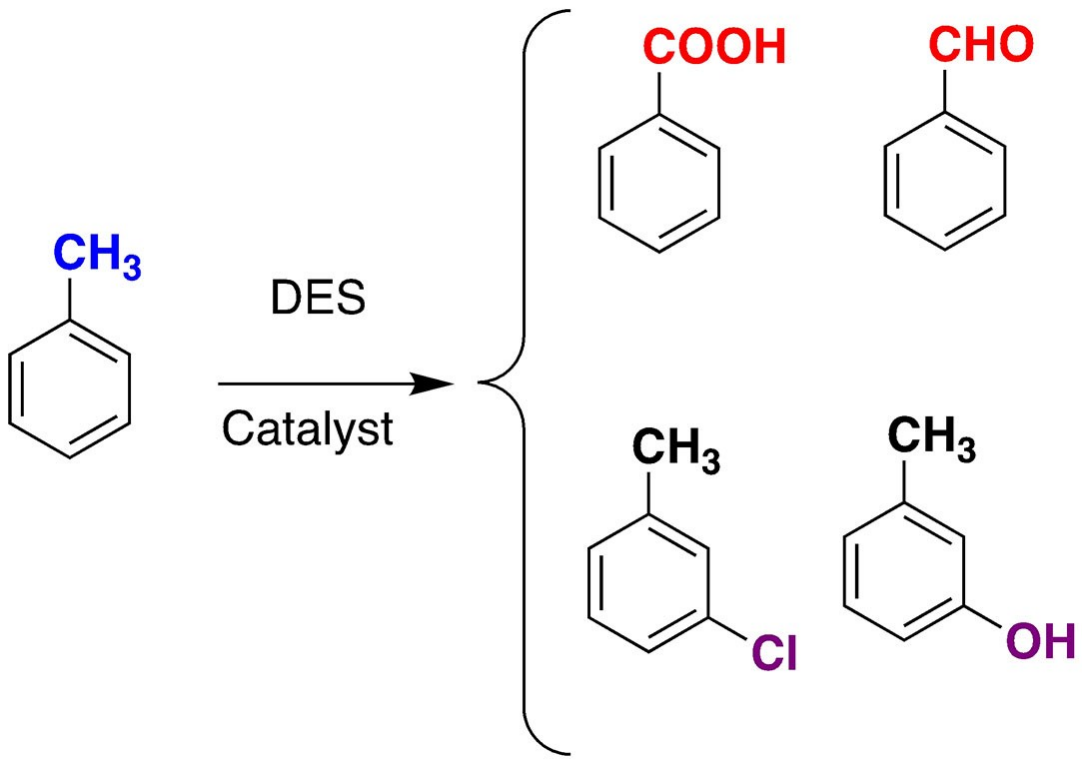
Oxidation of toluene with H_2_O_2_ in DES.

Green oxidation of toluene may be performed by hydrogen peroxide and several catalysts choline chloride based deep DES.^[120]^ Depending on reaction conditions, or catalist, several products may be obtained (Scheme 4).

A remarkable area related to Green Chemistry is the preparation of high value syntons from renewable sources as a real alternative to the petrochemical industry.[^121^, ^122^, ^123^] In this DES can play a relevant role, both as a solvent for extraction and reaction media for biomass derivatives transformation. As example on this area it can be described these two examples of oxidative transformations:

- The furfural conversion to maleic acid and fumaric acid in DES formed from oxalic acid and choline chloride (ChCl)[^119^, ^124^] both are valuable molecules, used for example in the in the synthesis of polymers (Scheme 5
  **Scheme 5:**
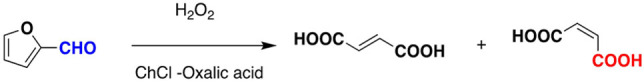
Fumaric and maleic acids from furfural.
  ).
- The transformation of cellulose into gluconic acid with gluconic acid self‐precipitation in a family of FeCl_3_ ⋅ 6H_2_O based catalytic deep eutectic solvents^[125]^ (Scheme 6
  **Scheme 6:**
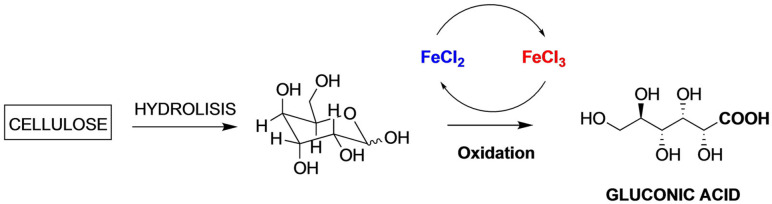
Gluconic acid from cellulose.
  ).

**Scheme 7:**
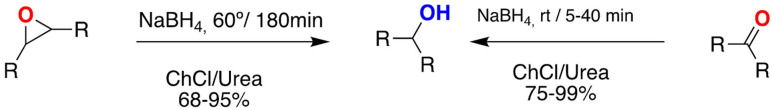
Reduction of epoxides and carbonyl compounds.

Reduction reactions can be performed in DES on a variety of substrates and reactives. Sodium borohydride reduction of carbonyl compounds and epoxides may be carried out with a green protocol for the regioselective and chemoselective transformation of epoxides and carbonyl compounds, in choline chloride/urea with good to excellent yields^[126]^ (Scheme 7).

**Scheme 8:**
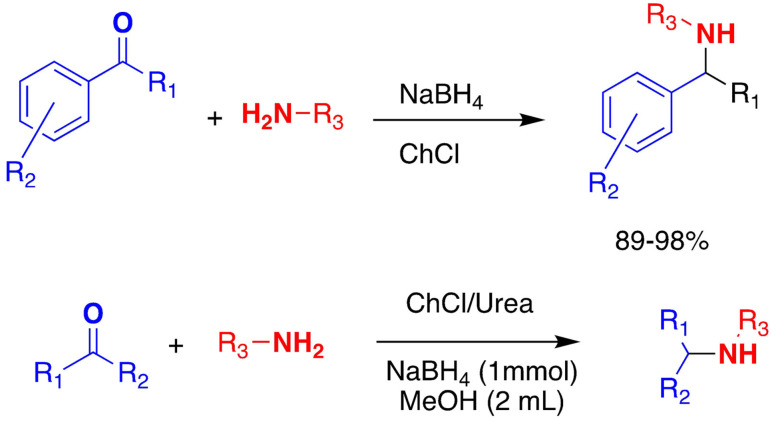
Reductive amination of aromatic carbonyl compounds.

Very similar conditions procure the reductive amination of carbonyl compounds Two examples are shown in Scheme 8 for aromatic carbonyl compounds and aldehydes.

The reductive amination of aldehydes in ChCl/Urea takes place with sodium borohydride as a reducing agent to give secondary amines with high selectivities and good yields.^[127]^

### 6.2 Hydrogenations

**Scheme 9:**
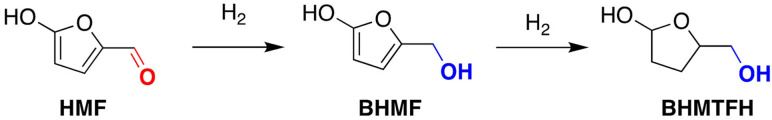
Hydrogenation of hydroxymethyl furfural.

Some work has been done, using DES for hydrogenation reactions. For example, and effective approach for the selective synthesis 2,5‐bishydroxymethylfuran (BHMF) from 5‐hydroxymethylfurfural (HMF) by solvent‐free hydrogenation in a deep eutectic mixture consisting of HMF (75 wt %) and choline chloride (ChCl). Selectivity of such conversion goes up to 94.6 % over Raney Co at 100 °C. BHMF can be transformed into bishydroxymetyltetrahydofuran (BHMFHF) in a subsequent reduction in the reaction conditions^[128]^ (Scheme 9).

Choline‐based DES have been used for hydrogenation reactions with other strategies, such as microencapsulation of catalysts in the hydrogenation of unsaturated compounds with Pd‐CHCl : TA@SiO_2_ microcapsules.^[129]^

### 6.3 Carbamate Synthesis

**Scheme 10:**
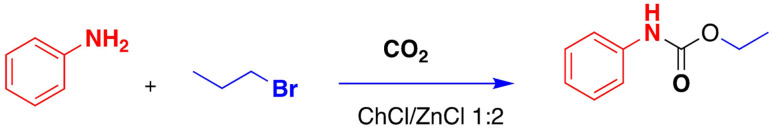
Carbamate synthesis in a DES.

DES has been used in the efficient synthesis of carbamates with carmon dioxide as reagent. This method is green and attractive solvent/catalyst system for the preparation of this family of compounds^[130]^ (Scheme 10).

### 6.4 Alkylations

**Scheme 11:**
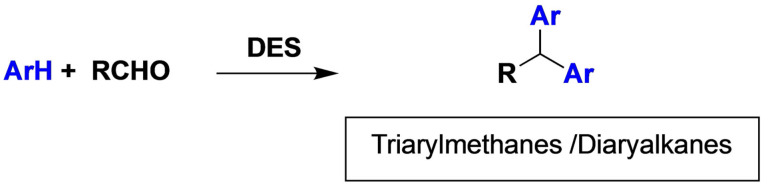
Alkylation on aromatic molecules.

The classic Friedel‐Crafts reaction has been carried out for the synthesis of triarylmethanes (TRAMs) and diarylalkanes (DIAAs) choline chloride‐zinc chloride ([ChCl][ZnCl_2_]_2_) based DES. The DES plays the role of solvent and Lewis acid and can be reused directly without any activation process five cycles, and the yield is in all cases upper than 89 %^[131]^ (Scheme 11).

### 6.5 Halogenations

**Scheme 12:**
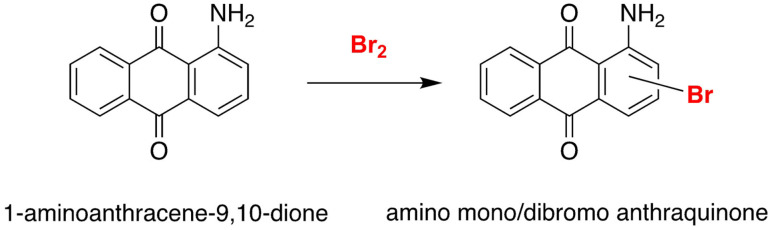
Bromination of 1‐aminoanthra‐9,10‐quinone.

As an example of halogenation reaction in a DES can be taken in account the bromination of 1‐aminoanthra‐9,10‐quinone (Scheme 12). It has eliminated any Volatile organic solvent and the use of concentrated acids like H_2_SO_4_, frequently employed as catalysts in these reactions using a simple ammonium deep eutectic solvent.^[132]^

### 6.6 Esterification and Trans‐Esterification

Several procedures for the esterification and transesterification reaction with DESs have been described in literature. These methods procure simple protocols, in many cases one of the components of DES is itself the necessary catalyst, and in many others the reaction media is compatible with the use of biocatalysts.

**Scheme 13:**
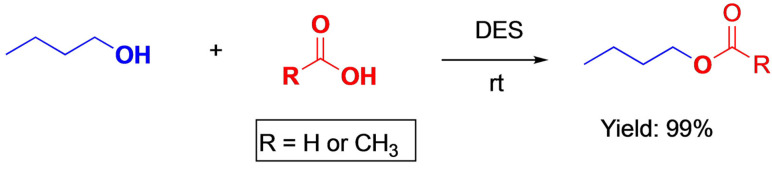
Esterification of acids in choline ChCl/Cr_3_Cl.6H_2_O.

Described below are three significant examples of esterification reactions using DES, one with a DES formed with metallic sal that procures acidic media, the other when DES delivers all the components necessary for the reaction, except carboxylic acid, and the last one, an example of biocatalysis for the reaction. The following examples show the potentiality of the DES application in this area.Formic and acetic acids are able to give esteres in the DES formed with choline chloride/chromium(III) chloride hexahydrate when mixed with alcohols at room temperature with good yields^[133]^ (Scheme 13).

**Scheme 14:**
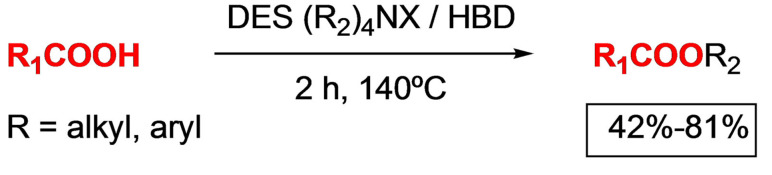
Esterification reaction with quaternary ammonium salt DES.

Other example of esterification reaction on carboxylic acids has been carried out with a quaternary ammonium salt of deep eutectic solvent (DES) which opens an access to esters using a family of DES as a solvent alkylating agent, and catalyst at the same time^[134]^ (Scheme 14).

**Scheme 15:**
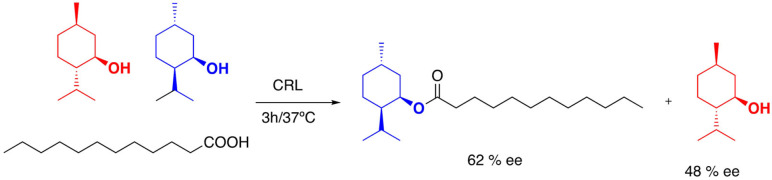
Enzymatic esterification of racemic menthol.

Because of the biocompatibility of many DESs, some experiments have been developed using enzymes in esterification and trans‐esterification reactions. Enzymatic esterification of racemic menthol with lauric acid in the presence of *Candida rugosa lipase* (CRL)reached up to 44 % after 3 h of reaction and an enantiomeric excess of the ester of 62 %^[135]^ (Scheme 15). Racemic menthol and lauric acid form in situ a DES that provides the separation of the mixture.

**Scheme 16:**
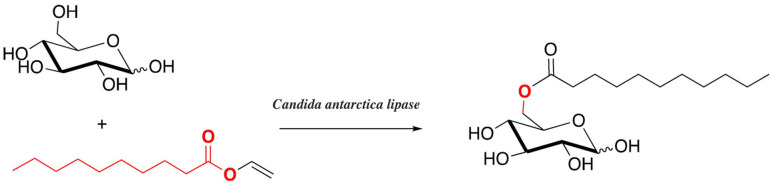
Transesterification reactions can be performed with DES.

Transesterification reactions can be performed with DES. As an example of such reactions are the synthesis of glycolipids. These products have paid attention in recent years as a way to prepare biodegradable surfactants. The enzymatic synthesis of glucose monodecanoate with two hydrophilic DES (choline:urea and choline:glucose) has been described in the presence of *Candida antarctica lipase* with vinyl decanoate^[136]^ (Scheme 16).

Transesterification reaction in DES has been used for the enzymatic biodiesel production from waste oils considering the procedure as an alternative to conventional production methods[^72^, ^137^, ^138^] in most cases focused on enzymatic reactions.

### 6.7 Condensations

C−C bond formation is a key strategy in organic synthesis because it lets us build complex molecules from simpler structures. DESs has also been used in these reaction types and a lot of work has been done about.^[139]^ Then, several examples will be described.

**Scheme 17:**
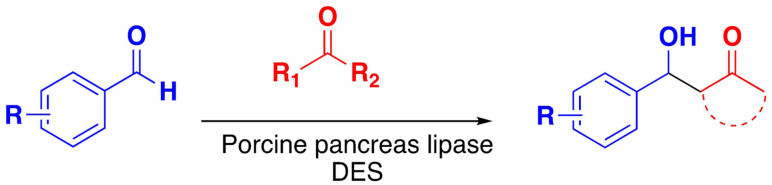
Enzymatic aldol condensation.

The well known aldol condensation reaction has been carried out on substituted benzaldehydes with aliphatic ketones in choline chloride/glycerol mixtures catalysed with *porcine pancreas lipase* (PPL) to give the aldol product high yields. DES shows excellent compatibility with PPL^[140]^ (Scheme 17).

DES was obtained from 3‐(cyclohexyldimethylammonio)propane‐1‐sulfonate and (1*S*)‐(+)‐10‐camphorsulfonic acid (SB3‐Cy/CSA) as a reaction media and catalyst for carbon–carbon bond formation reaction *via* Claisen–Schmidt condensation.^[141]^

**Scheme 18:**
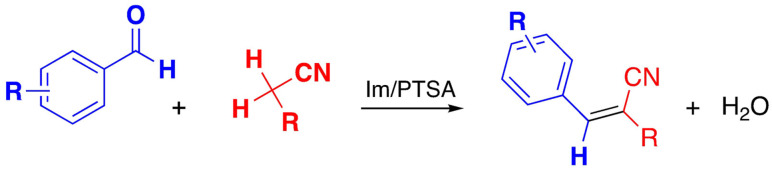
Knoevenagel condensation between aromatic aldehydes and active methylene compounds.

Another example of condensation reaction with DESs has been developed. by Qin et al. in an halogen‐free deep eutectic solvent (DES) for the well known Knoevenagel condensation between aromatic aldehydes and active methylene compounds at room temperature^[142]^ using as DES imidazole (Im) and p‐toluenesulfonic acid (PTSA) (Scheme 18).

### 6.8 Nucleophilic Substitutions

**Scheme 19:**
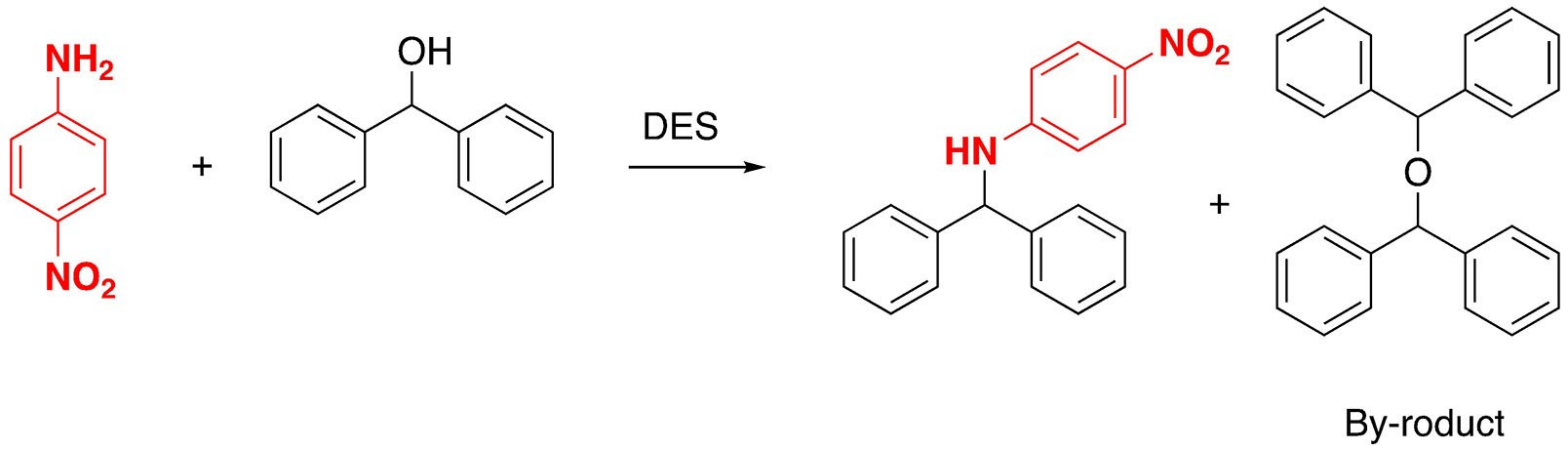
Nucleophilic substitution in ChCl/ ZnCl_2_.

Nucleophilic substitution is another classic reaction in organic synthesis that can be performed with DES. A convenient approach to the direct nucleophilic substitution reactions of alcohols, has been described using ChCl and ZnCl_2_ in a molar ratio 1 : 2 that as in other cases play as dual role of solvent and catalyst to obtain the final products with excellent isolated yields of 95 % (Scheme 19).

### 6.9 Diels‐Alder Reactions

**Scheme 20:**
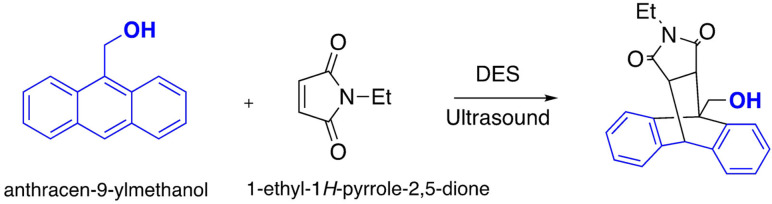
Example of Diels‐Alder reaction in DES.

Several DESs have been tested for the Diels–Alder reaction, using *N*‐ethylmaleimide as dienophile and changing the nature of the diene under both conventional heating and ultrasonic activation. Using ultrasonic activation in combination with DES proved good yields in drastically reduced reaction times^[143]^ (Scheme 20).

### 6.10 Cyclizations

**Scheme 21:**
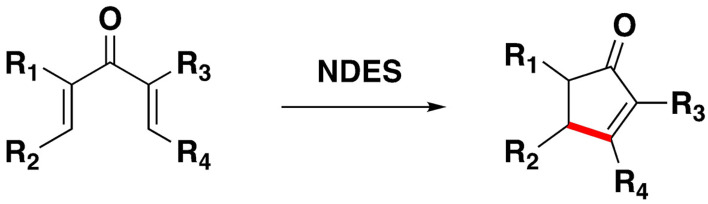
Cyclization with NDES.

Ciclization reactions have been developed with DES as reaction media. The Nazarov cyclization of the model substrate 2,4‐dimethyl‐1,5‐diphenylpenta‐1,4‐dien‐3‐one has been optimized in deep eutectic mixtures containing triphenylmethylphosphonium bromide (TPMPBr) and acetic acid or ethylene glycol as hydrogen bond donors.^[144]^ This reaction has been extended using NDES formed from choline chloride and carboxylic acids^[145]^ (Scheme 21).

### 6.11 Glycosylations

DES have been used as reaction media for the synthesis of alkyl glycosides catalyzed by thermostable α‐amylase from Thermotoga maritima Amy A. The enzyme was almost completely deactivated when assayed in a series of pure DES, but as cosolvents, DES containing alcohols, sugars, and amides as hydrogen‐bond donors (HBD) performed best.^[146]^

### 6.12 Peptide Synthesis

DESs have proved to be a good alternative to traditional organic solvents and in many biocatalytic processes,^[147]^ including enzymatic peptide synthesis. An example of enzymatic peptide synthesis is the choline chloride based DES as a neoteric solvent.^[148]^ An indirect procedure has been proposed for prebiotic replication of nucleic acids, and here are studied as media for prebiotic translation using NATS as a model. the storage of DNA‐conjugated activated esters in a DES followed by transfer into aqueous buffer to activate the NATS of peptides „on demand“. These findings, together with the reported functions of DESs in prebiotic processes.[^147^, ^149^]

### 6.13 Polymerization

DES may be used in polymer chemistry with three roles.^[150]^

- As a reaction media in polymerization reactions.
- As monomers in the synthesis of copolymers.^[151]^
- As media for modification a preexisting polymer.

An early example of polymerization using DES as reaction media was published in the synthesis of polyaniline polymer by electrochemical polymerization in an inorganic DES mixture formed by NH_4_F‐HF.^[152]^ Some examples about this methodology can be found in literature.[^146^, ^153^]

### 6.14 Multicomponent Reactions

A domino reaction is a process involving two or more bond‐forming transformations (usually C−C bonds) which take place under the same reaction conditions without adding additional reagents and catalysts, and in which the subsequent reactions result as a consequence of the functionality formed in the previous step.^[154]^

Multicomponent Reactions (MCRs) are considered as a subclass of domino reactions, one in which three or more molecules react to form a single product in a one pot reaction.[^154^, ^155^] They are an important tool in organic synthesis to obtain complex structures in one pot reaction, often used for the preparation of chemical libraries of functionalized heterocycles[^156^, ^157^] or heterocycles based scaffolds^[158]^ throw combinatorial chemistry processes.Under the perspective of green chemistry MCRs are specially convenient because of their high selectivity, and excellent atom‐economy.^[159]^ Sustainability of these reactions may be improved when DES are used as reaction media. In many cases the solvent acts as an acidic catalyst.

The classic Biginelli, Ugi and Passerini multicomponent reactions have been carried out using DES and some examples can be found in literature.

**Scheme 22:**
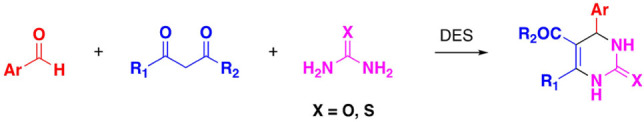
Biginelli reaction in DES.

Biginelli is a three‐component reaction between an aldehyde, a ß‐ketoester and urea with acid catalysis. This reaction has been performed with several mixtures of HBD and HBA as reaction media in the synthesis of 3,4‐dihydropyrimidin‐2(1H)‐one and thiones derivatives[^160^, ^161^] (Scheme 22).

**Scheme 23:**
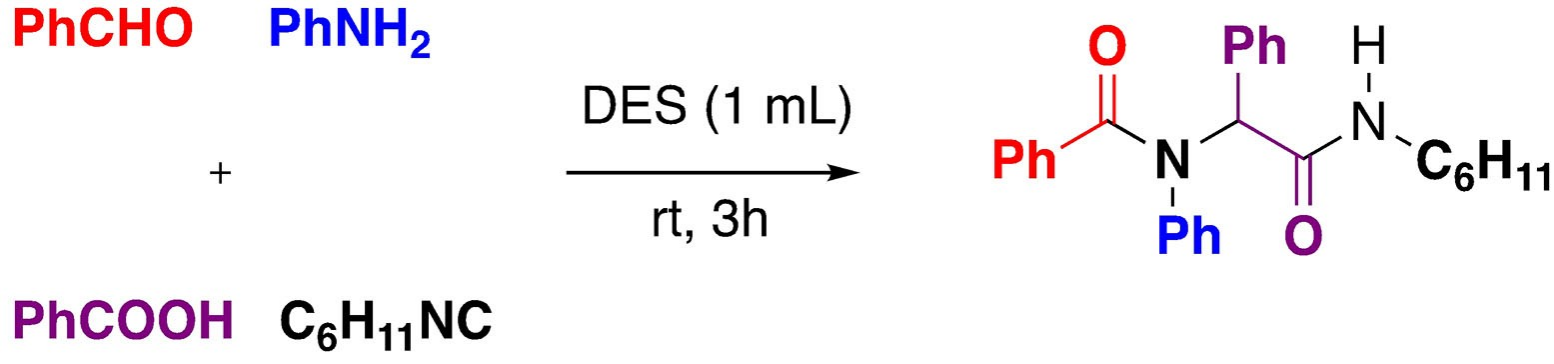
Ugi reaction.

Ugi is a four‐component reaction that has been extensively investigated for the preparation of compounds with pharmaceutical interest. Such reaction can be carried out for example with Choline chloride/Urea showing higher yields than conventional solvents^[162]^ (Scheme 23).

**Scheme 24:**
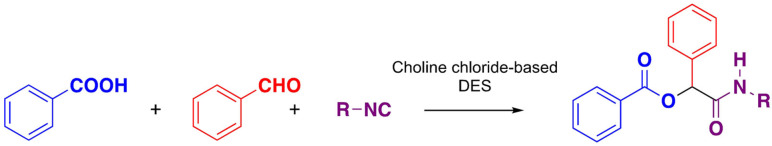
Passerini reaction.

The synthesis of α‐acyloxyamides may be performed with Passerini reaction. The α‐acyloxycarboxamide scaffold is present in many natural products as well as in pharmacologically active substances. This reaction can be accomplished in various choline chloride‐based deep eutectic solvents, mixing benzaldehyde, benzoic acid and cyclohexyl isocyanide in 1 : 1 : 1 proportions, without using any catalysts afforded products in excellent yield^[163]^ (Scheme 24).

**Scheme 25:**
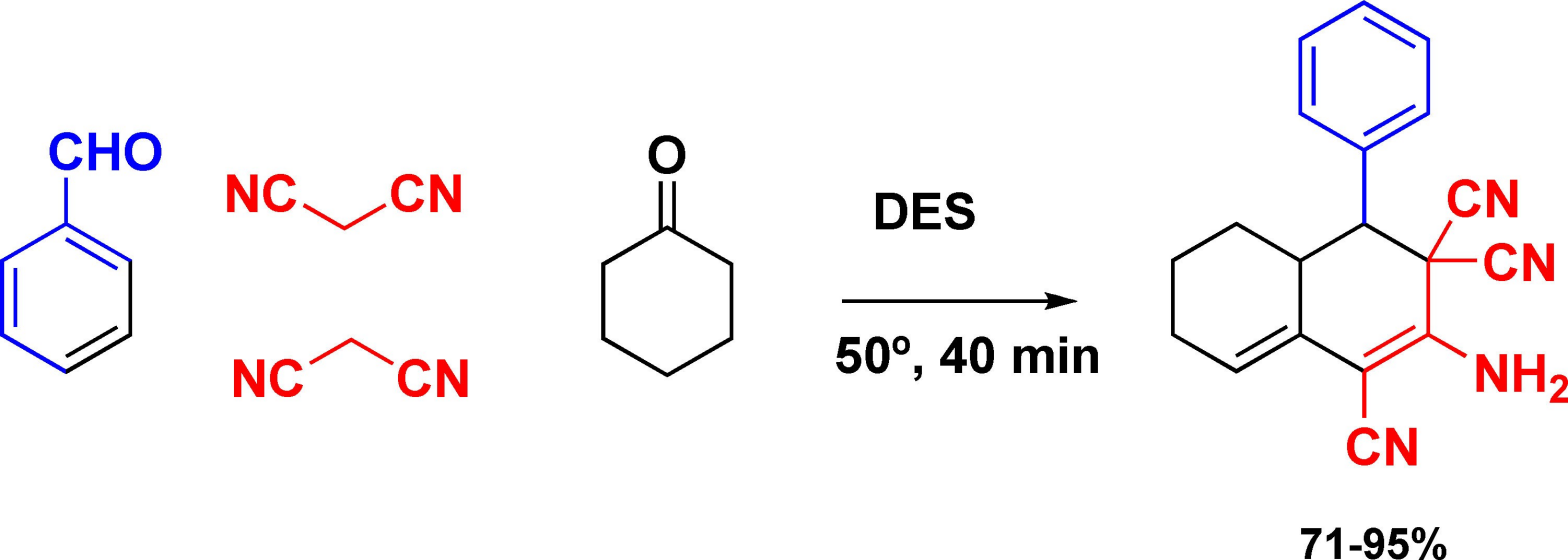
Example of multicomponent domino reaction.

The multicomponent domino reactions of ketones, aldehydes and malononitrile in a deep eutectic solvent (DES) were developed. Urea‐choline chloride based DES is effective dual solvents/organocatalysts and affording a series of multisubstituted cyclohexa‐1,3‐dienamines^[164]^ (Scheme 25).

## 7 Conclusions

Due to demands for greener technologies, the substitution of conventional solvents for others less toxic and with lower environmental impact has become a priority objective for scientific and manufacturing communities. Deep eutectic solvents may be considered as a valuable alternative because they can be easily prepared from affordable starting materials, reusable in most cases and many of them are completely biodegradables. They can be used in many fields as a valuable substitutes of conventional organic solvents to avoid common inconveniences for the separation or extraction of high value natural products, and as reaction media for many types of chemical transformations. Chemists can replace satisfactory, organic solvents produced by the petrochemical industry by deep eutectic solvents in conventional reaction conditions, or when required with ultrasound and microwave irradiation. A remarkable group of deep eutectic solvents are prepared from renewable sources, therefore they are biocompatible so they may be used in enzymatic transformations and show a promising future in pharmacology and medicine applications. Literature shows the unstoppable growth of publications that use deep eutectic solvents, so they can be taken into account as a valuable tool in green chemistry. In this review are described some of the leading fields when deep eutectic solvents may be applied in the present and with promising perspectives for the next years

## Conflict of interest

The authors declare no conflict of interest.

## Biographical Information

*Dr. Francisco G. Calvo‐Flores is Associate Professor of Chemistry in the Department of Organic Chemistry (University of Granada, Spain). He has developed its career at the University of Granada and during the last years he has been involved in several project focused on Green and Environmental Chemistry*.

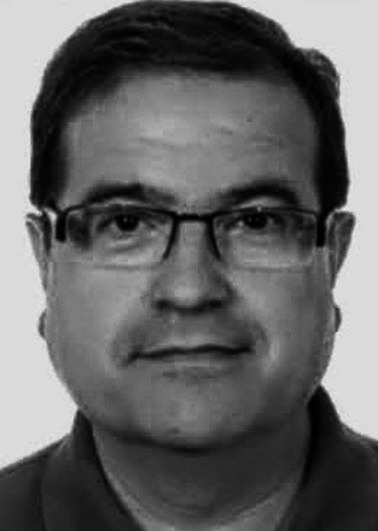

## Biographical Information

*Cristina Mingorance‐Sánchez studied chemsitry at the University of Granada, finishing the Degree in Chemistry in 2020. After her graduation, she started working in a fruit and vegetable company as a Quality Control Assistant, on sampling control, in accordance with the principles of the Hazard Analysis Critical Control Point (HACCP) of different food safety standards, developing effective self‐control systems*.

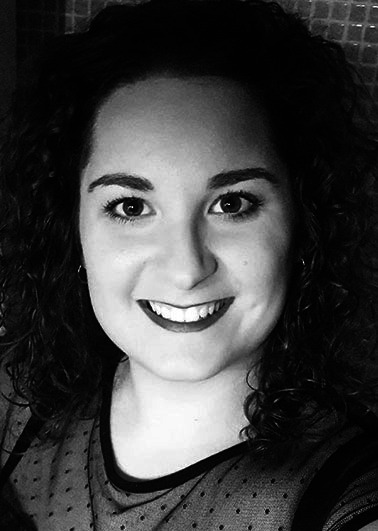

## Supporting information

As a service to our authors and readers, this journal provides supporting information supplied by the authors. Such materials are peer reviewed and may be re‐organized for online delivery, but are not copy‐edited or typeset. Technical support issues arising from supporting information (other than missing files) should be addressed to the authors.

Supporting Information
