# Supplementary material for: Deep Eutectic Solvents and Multicomponent Reactions: Two Convergent Items to Green Chemistry Strategies
Source: ChemistryOpen. 2021 Aug 17;10(8):815–29. doi: 10.1002/open.202100137 (PMC8369850; doi:10.1002/open.202100137)
Supplement: Supplementary file 1 — Supporting Information [file OPEN-10-815-s001.pdf]

Author Contributions

F.G.-F. Conceptualization:Lead; Supervision:Lead; Writing – original draft:Lead
